# Spinal intradural extramedullary cavernoma: A case report

**DOI:** 10.1016/j.ijscr.2024.109274

**Published:** 2024-01-13

**Authors:** Nahar Ismaiel, Hayyan Ibrahem, Georges Jabbour, Mohamad Joha, Mohammed Abdulrahman, Zuheir Alshehabi

**Affiliations:** a Cancer Research Center, Tishreen University, Latakia, Syria; b Department of Neurosurgery, Tishreen University Hospital, Latakia, Syria; c Department of Pathology, Tishreen University Hospital, Latakia, Syria; d Faculty of Medicine, Tishreen University, Latakia, Syria

**Keywords:** Intradural extramedullary cavernoma, Laminectomy, Case report

## Abstract

**Introduction:**

Cavernomas are rare vascular lesions that can occur anywhere along the neuraxis. However, they are most commonly found in the cerebral hemispheres. Spinal cavernomas are more uncommon and intradural extramedullary cavernomas are the most uncommon as they constitute only 3 % of spinal cavernomas.

**Presentation:**

A 36-year-old female presented to our neurosurgical clinic with a history of back pain radiating to the left side of the chest with left lower extremity paresthesia and ataxia without urinary disturbance. Neurological exam showed left-sided hypoesthesia below the T9 dermatome in addition to increased patellar and Achilles reflexes on the left side. MRI showed a homogeneous intradural extramedullary mass which was hyperintense on T1 and hypointense on T2 and it was surgically resected. Pathological examination confirmed the diagnosis of intradural extramedullary cavernoma.

**Discussion:**

Intradural extramedullary cavernomas are extremely rare lesions that arise within the area located between the inner surface of the dura and the pial surface of the spinal cord. Up until 2022 only 40 cases of intradural extramedullary cavernomas were reported in the literature. MRI is the preferred imaging modality and pathology is the golden standard for diagnosis. Surgical resection showed very promising results and it is considered the golden standard for treating this condition. However, surgery should be performed urgently to give an optimal outcome.

**Conclusion:**

Clinicians should consider this condition in their differential diagnoses when faced with progressive spinal root compression symptoms, sudden onset myelopathy, or progressive subarachnoid hemorrhage.

## Highlights

• Cavernomas are rare developmental malformations of the vascular bed that can be found most commonly in the cerebral hemispheres.
• Only a fraction of intradural spinal cavernomas are extramedullary as only 40 cases have been reported in the literature.
• The main imaging modality for intradural extramedullary cavernomas is Magnetic Resonance Imaging (MRI)
• Surgical resection should be performed urgently to halt any further neurological deterioration.
• Clinicians should consider this condition when faced with progressive spinal root compression or sudden onset myelopathy

## 1 Introduction

Cavernomas (sometimes referred to as cavernous hemangiomas or cavernous malformations) are rare developmental malformations of the vascular bed that can occur anywhere along the cerebrospinal axis but are most commonly found in the cerebral hemispheres [[1], [2], [3]]. Spinal cavernomas on the other hand are more uncommon with an incidence rate of around 5 % of all spinal vascular malformations [1].

The most common symptoms of spinal cavernomas are back pain accompanied by signs of progressive nerve root compression; however, sudden onset myelopathy and autonomic dysfunction accompanied by malaise were also reported [4].

Only 3 % of spinal cavernomas are located intraduraly [4,5] and only 40 cases of intradural spinal cavernomas are reported to be extramedullary [1,4]. Here we report a case of an intradural extramedullary cavernoma which is located at the T8-T9 level making it the 41st reported case in the literature. Finally, this case report has been reported in line with the SCARE criteria [6].

## 2 Presentation

A 36-year-old female presented to the neurosurgical clinic at our hospital with a 3-month history of back pain radiating to the left side of the chest in addition to left lower extremity paresthesia and ataxia without urinary disturbance. According to the patient, she experienced a gradual onset of low back pain without any previous trauma or muscle overload. She was treated with NSAIDs, analgesics, and muscle relaxants for 3 weeks without any improvement.

Neurological examination revealed left-sided hypoesthesia below the T9 dermatome. Lower extremity strength was normal with increased patellar and Achilles reflexes on the left side associated with extensor plantar responses. Laboratory tests were ordered (CBC, liver, and kidney panel) and were all within normal range.

**Fig. 1:**
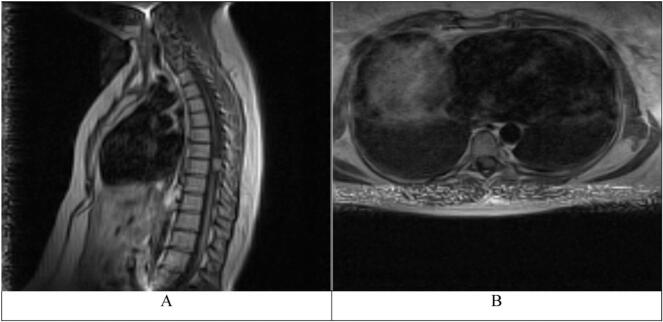
A. T1 weighted MRI, sagittal view. Hyperintense intradural extramedullary mass located at the T8-T9 level. B. T1 weighted MRI, axial view. The mass can be seen compressing the spinal cord anterolaterally.

A Magnetic Resonance Imaging (MRI) scan (Fig. 1) of the thoracic spine was ordered and it showed a homogeneous intradural extramedullary mass compressing the spinal cord at the T8-T9 level. The lesion was hyperintense on T1 and hypointense on T2-weighted images. The medical team decided to perform surgery to excise the lesion.

**Fig. 2:**
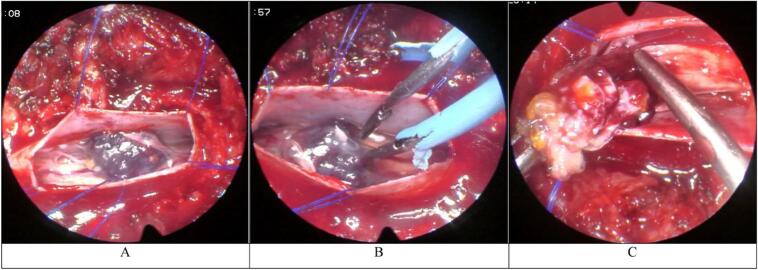
A. Surgical endoscopic view. Duratomy was achieved, the tumor is visible. B. Surgical endoscopic view. The tumor is cauterized using bipolar electrocautery, and the adhered nerve root is dissected free. C. Surgical endoscopic view. The tumor is resected en bloc.

Under general anesthesia, a T8-T9 laminectomy without fusion was performed (Fig. 2), and a linear duratomy was achieved.

The dorsal arachnoid appeared opaque and adhered to the rootlets. With endoscopic assistance, the tumor's feeding artery was cauterized using bipolar electrocautery.

A spinal nerve root was found to be adhered to the tumor thus it was successfully dissected free from the tumor using blunt dissection. The tumor was subsequently resected en bloc using tumor forceps and specimens were sent for pathological examination.

The duratomy was closed in a water-tight fashion, an adequate Redovac drain was placed in the cavity and the wound was closed in multiple layers. The drain was removed on post-op day 1.

A post-operative MRI scan was done and it showed complete resection of the tumor.

Postoperatively the patient's condition improved drastically and her symptoms subsided. She was able to return home several days later without any complications.

**Fig. 3:**
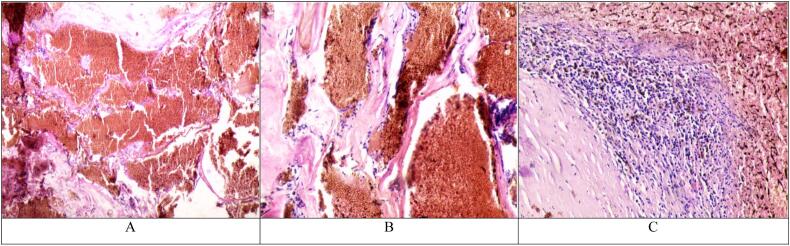
A. Intradural extramedullary cavernoma (H&E 40×). Nodular proliferation of dilated blood vessels with interstitial hemorrhage. B. Intradural extramedullary cavernoma (H&E 200×). Dilated blood vessels with abnormally thin walls lined with a single layer of flat endothelium. C. Intradural extramedullary cavernoma (H&E 100×). Hemosiderin deposition can be seen.

Gross examination of the specimen revealed a well-defined mass measuring approximately 1.5 cm, gray-brown in color and soft to rubbery in consistency. Microscopic examination (Fig. 3) showed nodular proliferation of dilated blood vessels intermixed with delicate fibrous connective tissue. The walls of these vessels are abnormally thin and are lined with a single layer of flat endothelium. Multiple foci of hemosiderosis were observed and no cellular atypia was noted. These findings confirmed the diagnosis of intradural extramedullary cavernoma.

## 3 Discussion

Spinal cavernomas are rare, accounting for 5–12 % of all spinal vascular anomalies [3]. The majority of spinal cavernomas arise from the vertebral body or the epidural spaces [7]. However, only 3 % of spinal cavernomas are located intraduraly and only a fraction of intradural spinal cavernomas are extramedullary as only 40 cases have been reported in the literature [4].

The pathogenesis of these uncommon lesions remains unknown [4]. None the less, some studies have suggested that these lesions can occur as a result of impaired migration and differentiation of the fetal mesoderm from the embryonic mesodermal plate during angioblastic differentiation [5].

Intradural extramedullary spinal cavernomas usually arise from the blood vessels surrounding the nerve roots, the inner layer of the dura mater, or the pial surface of the spinal cord [2,4]. They are found most commonly in the lumbar region, followed by the thoracolumbar junction, lower thoracic region, and the cervical region [8].

The clinical presentation of spinal cavernomas is varied. However, sensory dysfunction was the most common clinical manifestation which was followed by motor dysfunction and back pain [1].

Other less common symptoms include: subarachnoid hemorrhage, sciatic pain, autonomic dysfunction, hydrocephalus, dizziness, and cognitive dysfunction [1].

We have searched the related literature extensively and to our knowledge, there are only 40 reported cases of intradural extramedullary cavernoma which makes our case the 41st case of intradural extramedullary cavernoma reported in the literature.

The main imaging modality for intradural extramedullary cavernomas is Magnetic Resonance Imaging (MRI) as these lesions have certain characteristics that differentiate them from other spinal cord tumors or vascular malformations on MRI [9].

Intradural extramedullary cavernomas appear on MRI as well-defined lesions in the spinal cord with parts of these lesions demonstrating hypointense areas on both T1 and T2 weighted images which can be attributed to the presence of calcifications or dense fibrocartilage [10]. However, selective T2 hypointensity can occur due to the presence of hemosiderin [10].

Finally, the presence of a core of mixed high and low signal intensity which is circumscribed by a border of hypointensity (due to the macrophage uptake of hemosiderin) on both T1 and T2 weighted images is highly indicative of cavernomas [2].

Although MRI is a helpful tool in diagnosing intradural extramedullary cavernomas, pathology remains the golden standard. Under the microscope, cavernomas appear as well-demarcated lesions consisting of irregular sinusoidal vascular spaces that are lined by a single endothelial layer [2,8].

The golden standard for treating intradural extramedullary cavernomas is surgical resection which showed overwhelmingly positive outcomes as total resection was achieved in the majority of reported cases and the neurological outcome was excellent in all but 5 cases [1,11].

Although cavernomas normally present with slow, progressive deterioration of the neurological state; they can sometimes compress the spinal cord severely which can manifest as a sudden onset of symptoms and rapid neurological deterioration. Therefore urgent surgical intervention is crucial to prevent this from happening and to achieve the optimal outcome for the patient [4].

## 4 Conclusion

Intradural extramedullary cavernomas are rare lesions that arise within the area located between the inner surface of the dura and the pial surface of the spinal cord. Herein we presented a rare case of intradural extramedullary cavernoma. MRI was the main imaging modality and pathological examination confirmed the diagnosis. Clinicians should consider this condition in their differential diagnoses when faced with progressive spinal root compression symptoms, sudden onset myelopathy, or progressive subarachnoid hemorrhage. Finally, surgical resection showed great results in treating this condition. However, it should be performed urgently to achieve the best possible outcome.

## Consent

Written informed consent was obtained from the patient for publication of this case report and accompanying images. A copy of the written consent is available for review by the Editor-in-Chief of this journal on request.

## Ethical approval

Given the nature of the article, a case report, no ethical approval was required.

## Funding

No funding was needed.

## Author contribution

All authors contributed to this manuscript.

Nahar Ismaiel: Writing - original draft, reviewing, and editing.

Hayyan Ibrahem: Writing - original draft, reviewing, and editing.

Georges Jabbour: Writing - original draft, reviewing, and editing.

Mohamad Joha: Supervision; reviewing and editing.

Mohammed Abd Alrahman: Supervision; reviewing and editing.

Zuheir Alshehabi: Supervision; final reviewing and editing.

## Guarantor

Prof. Zuheir Alshehabi.

## Research registration number

N/A.

## Provenance and peer review

Not commissioned, externally peer-reviewed.

## Conflict of interest statement

The authors declare no conflict of interest.
